# Presynaptic HCN channels constrain GABAergic synaptic transmission in pyramidal cells of the medial prefrontal cortex

**DOI:** 10.1242/bio.058840

**Published:** 2022-03-21

**Authors:** Wei Cai, Shu-Su Liu, Bao-Ming Li, Xue-Han Zhang

**Affiliations:** 1 State Key Laboratory of Medical Neurobiology and MOE Frontiers Center for Brain Science, Institutes of Brain Science, Fudan University, Shanghai 200032, China; 2 School of Basic Medical Science and Institute of Brain Science, Hangzhou Normal University, Hangzhou 311121, China

**Keywords:** GABAergic transmission, HCN channel, mPFC, Rats

## Abstract

Hyperpolarization-activated cyclic nucleotide-gated (HCN) channels are widely expressed in neurons in the central nervous system. It has been documented that HCN channels regulate the intrinsic excitability of pyramidal cells in the medial prefrontal cortex (mPFC) of rodents. Here, we report that HCN channels limited GABAergic transmission onto pyramidal cells in rat mPFC. The pharmacological blockade of HCN channels resulted in a significant increase in the frequency of both spontaneous and miniature inhibitory postsynaptic currents (IPSCs) in mPFC pyramidal cells, whereas potentiation of HCN channels reversely decreases the frequency of mIPSCs. Furthermore, such facilitation effect on mIPSC frequency required presynaptic Ca^2+^ influx. Immunofluorescence staining showed that HCN channels expressed in presynaptic GABAergic terminals, as well as in both soma and neurite of parvalbumin-expressing (PV-expressing) basket cells in mPFC. The present results indicate that HCN channels in GABAergic interneurons, most likely PV-expressing basket cells, constrain inhibitory control over layer 5–6 pyramidal cells by restricting presynaptic Ca^2+^ entry.

## Abstract (precis)

**Summary:** This study we uncover a novel function of HCN channels in layer 5–6 medial prefrontal cortex (mPFC) circuit: regulate inhibitory input of pyramidal neurons.

## INTRODUCTION

Hyperpolarization activated cyclic nucleotide-gated (HCN) channels are richly expressed in the central nervous system, which consist of four either identical or nonidentical subunits (HCN1-4) (Santoro and Tibbs, 1999), are activated with membrane hyperpolarization, and are regulated directly by cAMP (Baruscotti and Difrancesco, 2004; Biel et al., 2009; Robinson and Siegelbaum, 2003). HCN channels conduct a current called I_h_, which contributes to resting potential and input resistance. HCN channels have an important role in controlling neuronal intrinsic excitability, dendritic integration of synaptic potentials, synaptic transmission, and rhythmic oscillatory activity in individual neurons and neuronal networks (Robinson and Siegelbaum, 2003; Lorincz et al., 2002; Gauss and Seifert, 2000; Pape, 1996; Day et al., 2005; Notomi and Shigemoto, 2004).

HCN channels are principally located in the pyramidal cell dendrites, although they are found at lower densities in the soma of pyramidal neurons as well as other neuron subtypes (Shah, 2014). Somato-dendritic HCN channels in pyramidal neurons modulate spike firing and synaptic potential integration by influencing the membrane resistance and resting membrane potential (Shah, 2014). In addition to their dendritic localization, HCN channels are expressed in cortical and hippocampal axons and synaptic terminals of inhibitory and excitatory neurons (Lorincz et al., 2002; Notomi and Shigemoto, 2004; Bender et al., 2007; Boyes et al., 2007; Lujan et al., 2005). Additionally, presynaptic HCN current, I_h_, has been indicated to affect excitatory synaptic transmission in invertebrate neurons and vertebrate neurons where I_h_ has been shown to influence excitatory transmitter release (Beaumont et al., 2002; Beaumont and Zucker, 2000; Huang et al., 2011) via affecting the activities of presynaptic terminal Ca^2+^ channels (Huang et al., 2011). Additionally, presynaptic I_h_ affects inhibitory neurotransmission in the rodent globus pallidus, cerebellum and hippocampus (Boyes et al., 2007; Lujan et al., 2005; Aponte et al., 2006; Southan et al., 2000), though the mechanism by which these occur is unknown.

The prefrontal cortex (PFC) plays a critical role in cognitive functions. The PFC is composed of two major neuronal populations: glutamatergic pyramidal neurons and γ-aminobutyric acid (GABAergic) interneurons. Although GABAergic interneurons only account for approximately 20% of the cortical neuronal population, they are critical elements of cortical circuits by providing feedforward and feedback inhibition and generating synchronous and rhythmic network activity (McBain and Fisahn, 2001).

Although GABAergic interneurons are a minority of the neuron population in the PFC (10–20%), each interneuron could control hundreds to thousands of pyramidal cells through its profuse local axonal arborizations. Somatostatin, Calretinin, Parvalbumin (PV)-expressing basket cells comprise ∼50% of GABAergic neuron population in the neocortex (Gonchar and Burkhalter, 1997). Axons of PV-expressing basket cells preferentially target the soma and proximal dendrites of pyramidal cells, forming multiple inhibitory synapses with a high probability of GABA release (Gonchar and Burkhalter, 1997; Freund and Katona, 2007; Markram et al., 2004; Buhl et al., 1994). Thus, PV-expressing basket cells exert a powerful inhibitory control over pyramidal cells and are likely to constitute the dominant inhibitory system in the PFC.

It has been noted that peri somatic inhibition ensured by basket cells also has a powerful regulatory effect on the synchronization and oscillation of pyramidal cells (Freund and Katona, 2007; Bartos et al., 2007). Neuronal synchronization and oscillation are necessary for the execution of cognitive functions (Lisman et al., 2008), and abnormal synchronization and oscillation in the PFC may result in cognitive deficits seen in psychiatric disorders (Lisman, 2011). Wang et al. reported that the α_2A_ adrenoceptor-cAMP-HCN channel signaling pathway in the prefrontal cortical cells of monkeys plays an important role in maintaining the delay-period persistent firing, such as facilitating working memory, although the cell-type localization of HCN channels remains to be identified (Wang et al., 2007).

However, little is known about the role of HCN channels in GABAergic interneurons of the cortex. The present study attempted to examine whether and how HCN channels in interneurons regulate inhibitory synaptic transmission onto layer 5–6 pyramidal cells in the medial prefrontal cortex of rats, using immunofluorescence staining and whole-cell recording approaches.

## RESULTS

### HCN channels limit GABAergic transmission onto pyramidal cells

**Fig. 1:**
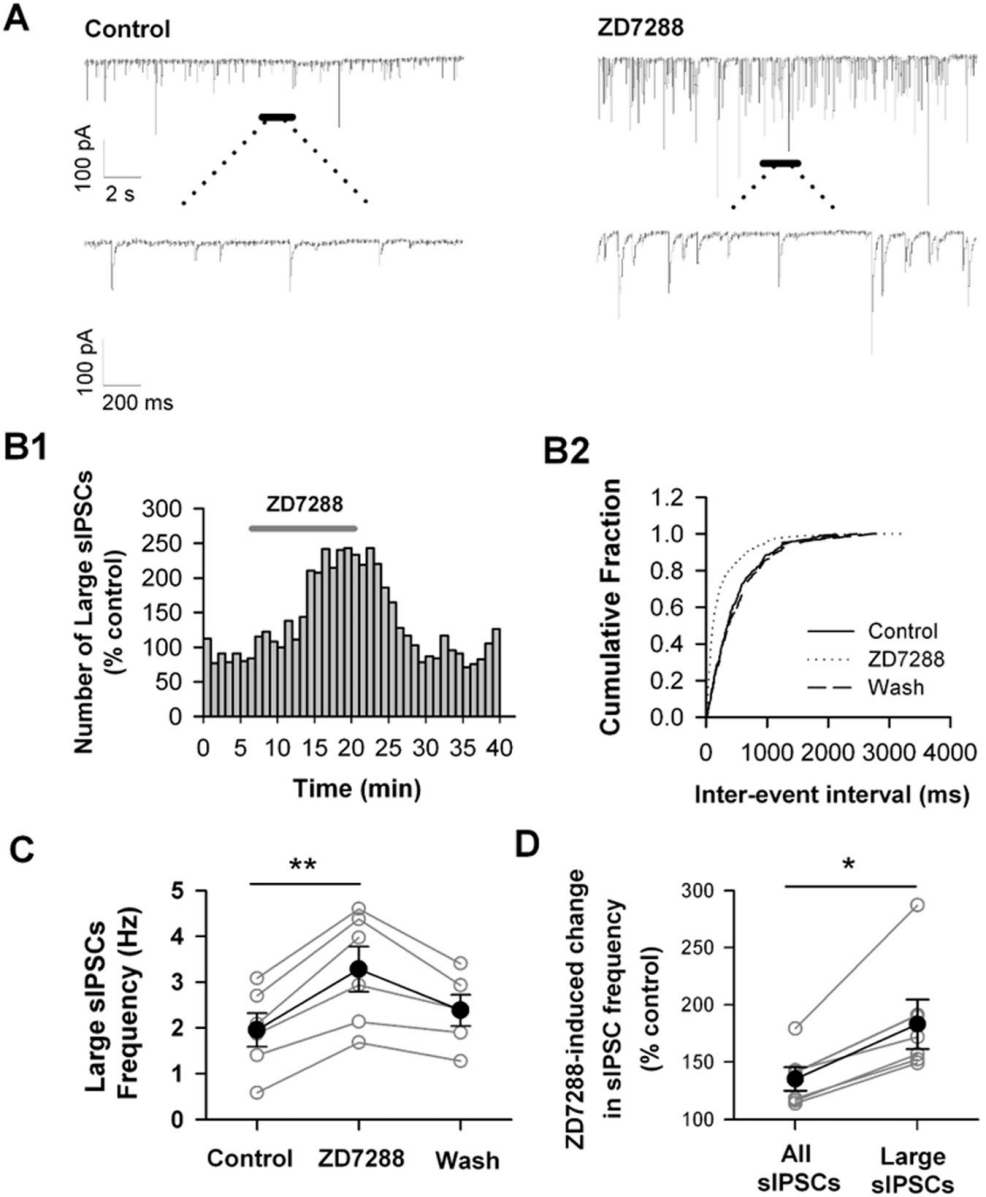
**. Blockade of HCN channels increases the frequency of sIPSCs in mPFC pyramidal cells.** (A) An example trace of sIPSCs recorded in mPFC pyramidal cell in absence (Control) or presence of HCN channel blocker ZD7288 (30 µM). Holding potential=−70 mV. (B) ZD7288 increases the frequency of sIPSCs with large amplitude (> 20 pA). The number distribution of large sIPSCs (bin=60 s; *B1*), and the cumulative fraction distribution of inter-event intervals of sIPSCs before (Control), during (ZD7288), and after ZD7288 application (Wash) (B2). Data were from the same cell in A. (C) The summary individual (open circles) and grouped (closed circles) frequency of large sIPSCs. *n*=6 cells. **P<0.01. (D) The sIPSC frequency in all detective events and in large events after ZD7288 application. Open circles for the individual cell; Close circles for grouped cells. Data were from the same cell in C. *P<0.05.

To examine whether HCN channels are involved in regulating GABAergic synaptic transmission, we recorded action potential-dependent spontaneous IPSCs (sIPSC) in PFC layer 5–6 pyramidal cells in the presence of 20 µM DNQX and 50 µM D-APV with −70 mV holding potential (Fig. 1A). The recorded sIPSCs could be completely blocked by the GABA_A_ receptor antagonist bicuculline (10 µM) (data not shown). Under the experimental control condition, the frequency of sIPSC, especially, large sIPSCs (amplitude >20pA) was 1.95±0.37 Hz, and it increased to 3.28±0.5 Hz 12–15 min after bath application of HCN channel blocker ZD7288 (30 µM) (Fig. 1C; *P*<0.01, paired *t*-test, *n*=6 cells). The facilitation effect of ZD7288 was largely reversible 12–15 min after termination of ZD7288 (Fig. 1C; 2.62±0.31 Hz after washout). The ZD7288-enhancement of the frequency of sIPSCs in pyramidal cells suggests that HCN channels limit GABAergic transmission onto pyramidal cells.

### Presynaptic but not postsynaptic HCN channels are involved in limiting GABAergic transmission

**Fig. 2.**
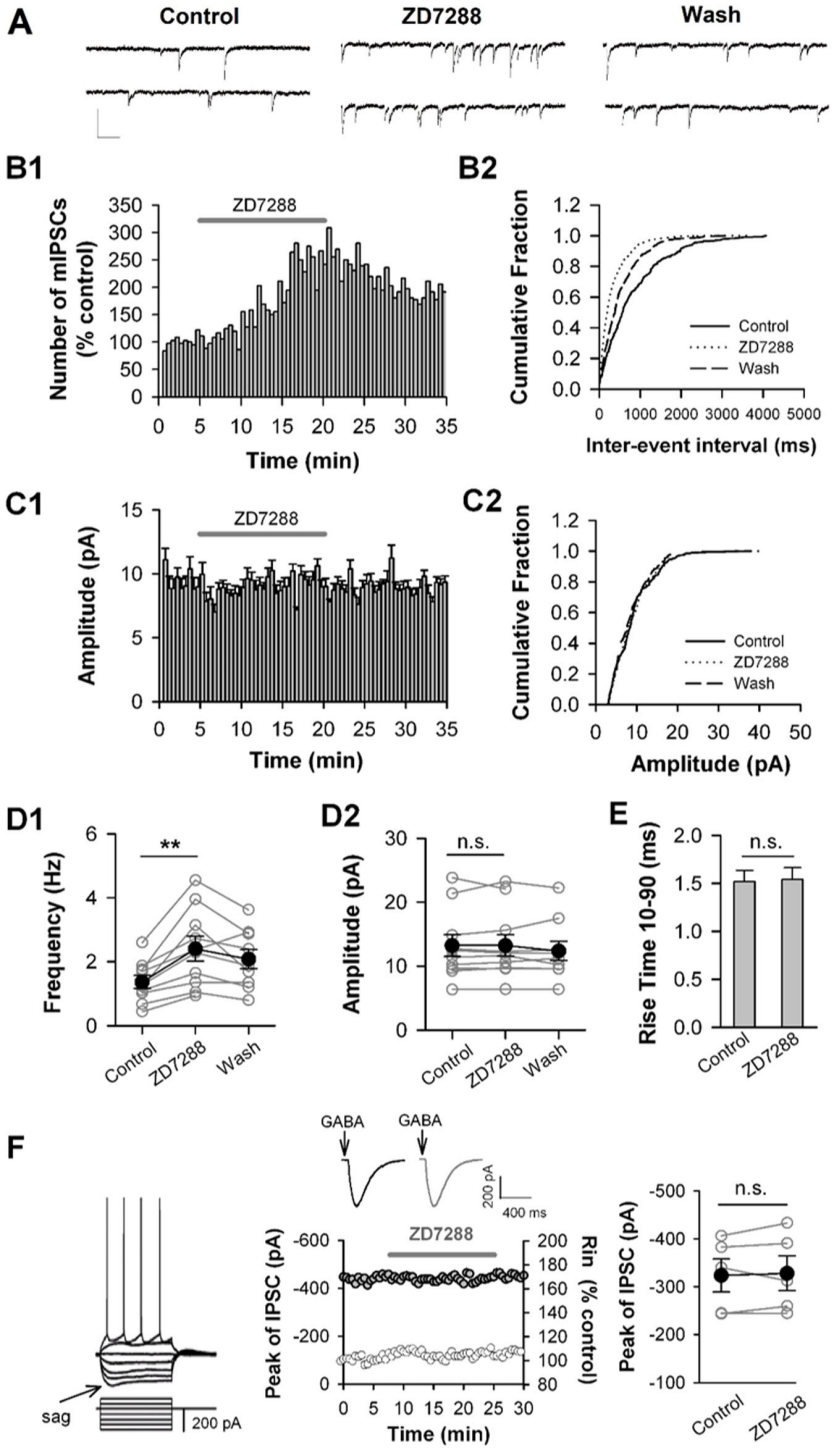
**Blockade of HCN channels enhances the frequency but not amplitude of mIPSCs.** (A) Representative traces of miniature IPSCs (mIPSCs) recorded in mPFC pyramidal cell before (Control), during (ZD7288), and after ZD7288 application (Wash). Holding potential: −70 mV. Calibration: 20 pA, 200 ms. (B) ZD7288 facilitates the frequency of mIPSCs. The number distribution of mIPSCs (bin=60 s, B1), and the cumulative fraction distribution of inter-event intervals of mIPSCs (B2). Data were from the same cell in A. (C) ZD7288 has no effect on the amplitude of mIPSCs. The amplitude distribution of mIPSCs (bin=60 s, C1), and the cumulative fraction distribution of mIPSC amplitude (C2). Data were from the same cell in A. (D) The mIPSC frequency (D1) and amplitude (D2) from the individual cell (open circles) and grouped cells (closed circles). *n*=10 cells, **P<0.01. (E) ZD7288 has no effect on 10–90% rise time of mIPSCs. Data were from the same cells in D. (F) ZD7288 has no effect on the amplitude of IPSCs evoked by puff application of GABA (10 µM) to pyramidal cells. A typical example for pyramidal cells with a sag (left). An example of time course of the IPSC amplitude (black circles) and the input resistance (grey circles) obtained from a pyramidal cell. The insets show the IPSC traces in the absence (black) and presence of ZD7288 (grey), each of which is the average of seven consecutive IPSCs (middle). The summary individual (open circles) and grouped (closed circles) amplitude of IPSCs (right). *n*=5 pyramidal cells.

To test how HCN channels affect GABAergic transmission by postsynaptic or presynaptic pathway, we examined miniature IPSCs (mIPSCs), which can represent responses of pyramidal cells to spontaneous release of single GABA-containing vesicles, and action potential independently. Therefore, mIPSCs were recorded in the presence of 1 µM tetrodotoxin (TTX) that blocks action potential firing and propagation. As shown in Fig. 2, the frequency of mIPSCs was 1.37±0.20 Hz and the peak amplitude was 13.21±1.73 pA under the control condition. When ZD7288 (30 µM) was applied, the frequency of mIPSCs increased to 2.40±0.39 Hz (Fig. 2D1; *P*<0.01, paired *t*-test, *n*=10 cells), whereas the amplitude of mIPSCs kept unchanged (Fig. 2D2; *P*>0.05, paired *t*-test). The facilitation effect of ZD7288 on mIPSC frequency was largely recovered after termination of ZD7288 application (2.08±0.30 Hz after washout; Fig. 2D1). The fast rise time of mIPSCs in the presence of ZD7288 was comparable with control (10–90% rise time: 1.52±0.12 ms under the control and 1.54±0.13 ms in the presence of ZD7288; *P*>0.05, paired *t*-test; Fig. 2E). Thus, ZD7288 did not alter the kinetics of mIPSCs. Together, mIPSC events regulated by ZD7288 were mainly generated in the presynaptic region of recorded pyramidal neurons (Soltesz et al., 1995). These results suggested that HCN channels may limit presynaptic GABA release to constrain GABAergic transmission onto pyramidal cells.

To further clarify whether HCN channels in postsynaptic pyramidal cells are involved in the ZD7288 facilitation effect on GABAergic transmission, we evoked postsynaptic GABA-receptor-mediated currents by puffing GABA onto the soma of pyramidal cells, and examined the effect of ZD7288 on GABA-evoked currents. To ensure the recorded pyramidal cells expressing HCN channels, we only examined the effect of ZD7288 on GABA-evoked currents in the pyramidal cells with depolarizing sag (Fig. 2F, left). It is well known that the depolarization sag in response to negative current injection is a typical response pattern of pyramidal cells expressing HCN channels in the PFC (Day et al., 2005) and other tissues (Maccaferri et al., 1993; Magee, 1998; Berger et al., 2001). GABA-evoked currents recorded in the presence of TTX (1 µM), DNQX (20 µM) and D-APV (50 µM) with −70 mV holding potential (Fig. 2F, middle, inset). Puff application of GABA (10 µM) evoked an outward current that was completely blocked by the GABA_A_ receptor antagonist bicuculline (10 µM; *n*=3; data not shown). As shown in Fig. 2F, neither the amplitude of GABA-evoked currents (Fig. 2F, right; Control: −323.59±34.04 pA; ZD7288: −328.10±36.71 pA), nor the input resistance of recorded pyramidal cells (Fig. 2F, middle; 108.55±3.10% of the control 12–15 min after ZD7288 application, *P*>0.05 for ZD7288 versus control, paired *t*-test) was altered by ZD7288. Therefore, the ZD7288-induced increase in the frequency of mIPSC was not due to the blockade of HCN channels in the pyramidal cells, but most likely resulted from the blockade of HCN channels in the GABAergic terminals innervating the pyramidal cells.

### HCN channels are present on presynaptic GABAergic terminals

**Fig. 3.**
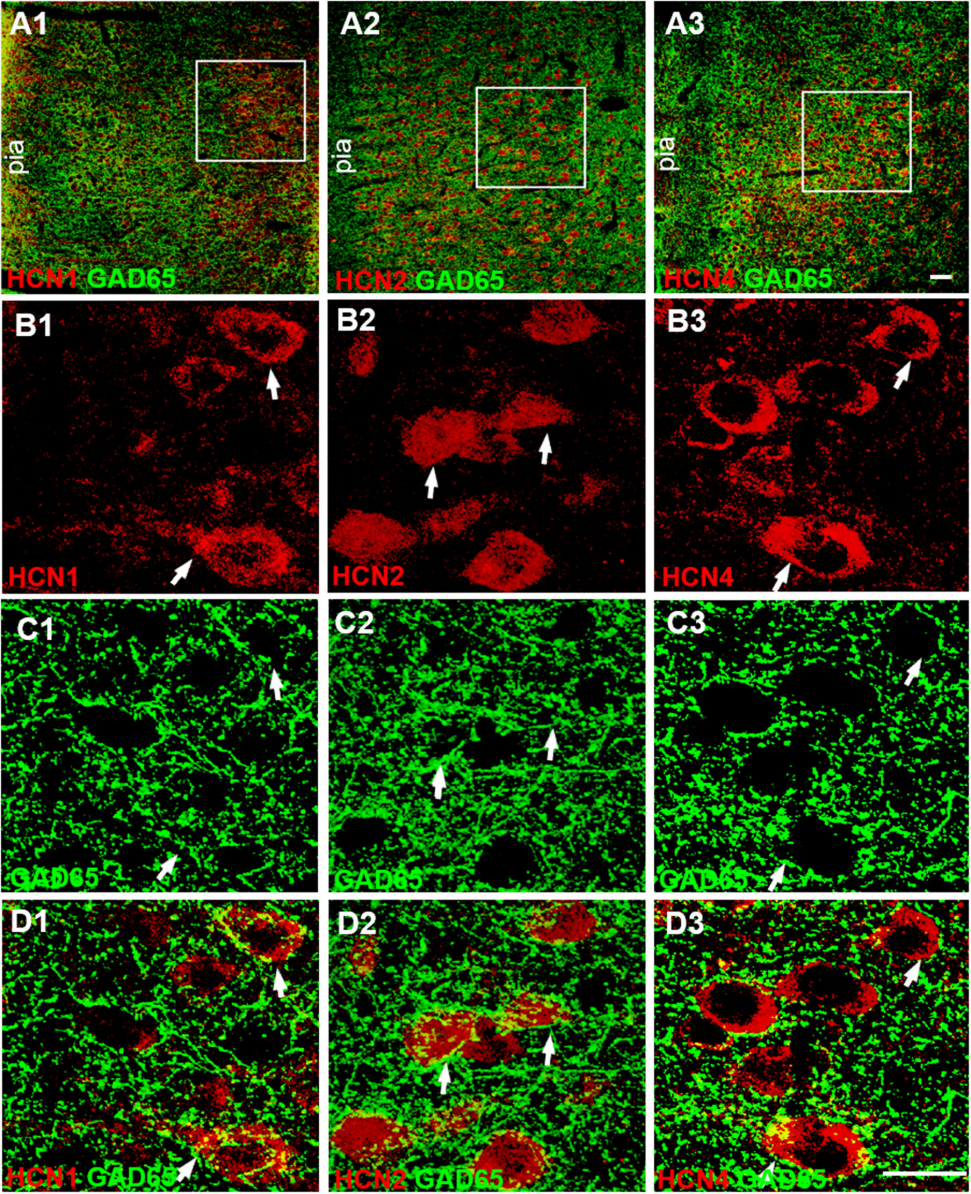
**HCN channels are present on GABAergic terminals in the mPFC.** (A) Low-magnification confocal images showing double stained with HCN channels (red) and GAD65 (green), a GABAergic terminal marker. The squares illustrate the cells in layers 5–6 of the mPFC. Scale bar: 40 µm. (B,C) Single-plane confocal images showing the HCN1-ir (B1), HCN2-ir (B2), HCN4-ir (B3), and GAD65-ir (C1-C3) at high magnification. GAD65-ir appears in punctuate structures distributed in the neuropil, as well as around unlabeled pyramidal cell soma (C1-C3). (D) Merging of the paired images (B1 and C1), (B2 and C2), and (B3 and C3) shows that the puncta of GAD65-ir surround the cell bodies of HCN1-ir (B1), HCN2-ir (B2), and HCN4-ir (B3) cells. Partially overlapping areas of red (HCN) and green (GAD65) profiles showing yellow. The arrowheads indicate double-labeled cells. Scale bar: 20 µm.

To identify the expression of HCN channels in presynaptic GABAergic terminals, we examined the expression of HCN channels in GABAergic terminals using immunostaining techniques. We performed immunolabeling against GAD65, the synthetic enzyme for GABA, to label GABAergic terminals (Fiorentino et al., 2009). We double labeled HCN1, HCN2, and HCN4 channels with GAD65. Single-plane confocal images showed that GAD immunoreactive (GAD-ir) appeared in puncta structures distributed in the neuropil, as well as around unlabeled cell bodies (Fig. 3C), which were consistent with previous reports (Schwab et al., 2013). Merging single-plane images showing HCN1-ir, HCN2-ir, and HCN4-ir with GAD65-ir showed the puncta of GAD65-ir, surrounding the cell bodies of HCN-ir cells, partially co-located with HCN1-ir, HCN2-ir, and HCN4-ir (Fig. 3D). These data underline that HCN channels are present in the GABAergic terminals, indicating that blockade of HCN channels affects presynaptic GABA release.

### HCN channel activation suppress GABA release

**Fig. 4.**
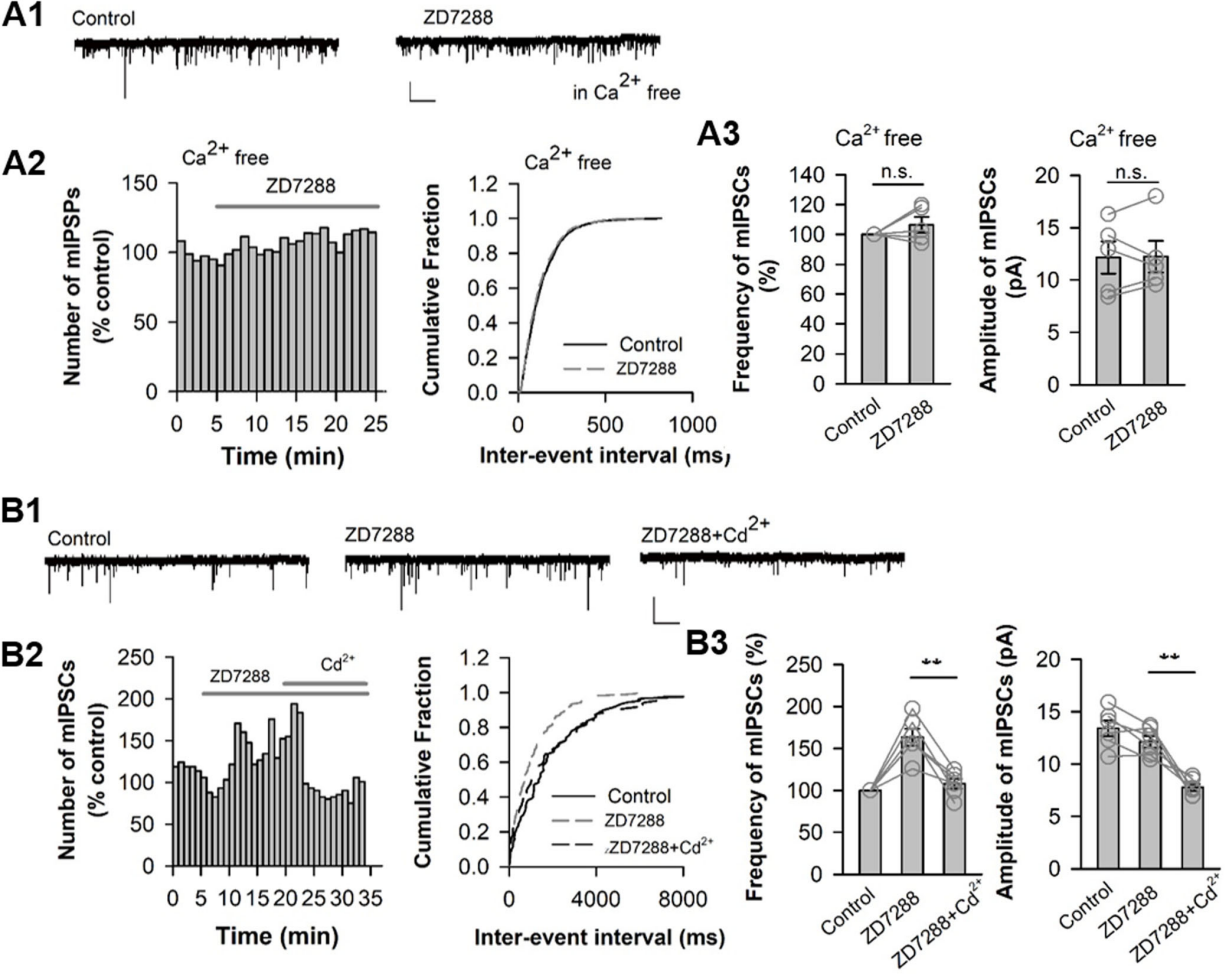
**Enhancing HCN channel function decreases the frequency of mIPSC.** (A) Representative traces of mIPSCs recorded in mPFC pyramidal cell before (Control), during (Sp-cAMPs), and after Sp-cAMPs application (Wash). Holding potential: −70 mV. Calibration: 1 s, 10 pA. (B) Effects of Sp-cAMPs on the frequency and amplitude of mIPSCs. The number distribution of mIPSCs (bin=60 s; B1), and the cumulative fraction distribution of inter-event intervals (B2) and amplitude (B3) of mIPSCs. Data were from the same cell in A. (C) Summary for individual cell (open circles) and grouped cells (closed circles). *n*=5 cells, **P<0.01.

Some researchers have pointed out that ZD7288 can activate Na+ and Ca2+ channels (Liu et al., 2009; Sanchez-Alonso et al., 2008). Thus, we ought to clarify this phenomenon is based on HCN channels. Activation of HCN channels is facilitated by cAMP (cyclic adenosine monophosphate). cAMP strongly enhances HCN channel function (Ludwig et al., 1998; Chen et al., 2001; Ulens and Tytgat, 2001; Wainger et al., 2001; Stieber et al., 2003). In the cortex, HCN channels are heteromers of HCN1 and HCN2 subunits that are highly responsive to cAMP (Chen et al., 2001; Ulens and Tytgat, 2001). We hypothesized that upregulation of presynaptic HCN channel function might alter GABA release onto pyramidal cells. To test this hypothesis, the frequency of mIPSC was compared after perfusion of the membrane-permeable, cAMP analog, Sp-cAMPs (200 µM). Perfusion of Sp-cAMPs (5 min) markedly decreased the frequency of mIPSCs in the pyramidal cell (Fig. 4B2; *P*<0.01) whereas it had no impact on the amplitude of mIPSC (Fig. 4B3; *P*<0.01, Kolmogorov–Smirnov test). Grouped data demonstrate that Sp-cAMPs (12–15 min after application) significantly decreased mIPSC frequency from 1.71±0.28 Hz to 1.26±0.24 Hz (Fig. 4C; *P*<0.01, paired *t*-test; *n*=5 pyramidal cells from two animals). The effect of Sp-cAMPs on mIPSC frequency was largely recovered after termination of Sp-cAMPs application (Fig. 4C1; 0.56±0.26 Hz after washout; *P*<0.05 versus Sp-cAMPs treatment; paired *t*-test). Sp-cAMPs had no impacts on the amplitude and kinetics of mIPSCs (Fig. 4C2,C3). This result solidified our hypothesis that ZD7288 influence mIPSC frequency through HCN channels.

### HCN-channel blockade facilitates GABA release via presynaptic Ca^2+^ influx

**Fig. 5.**
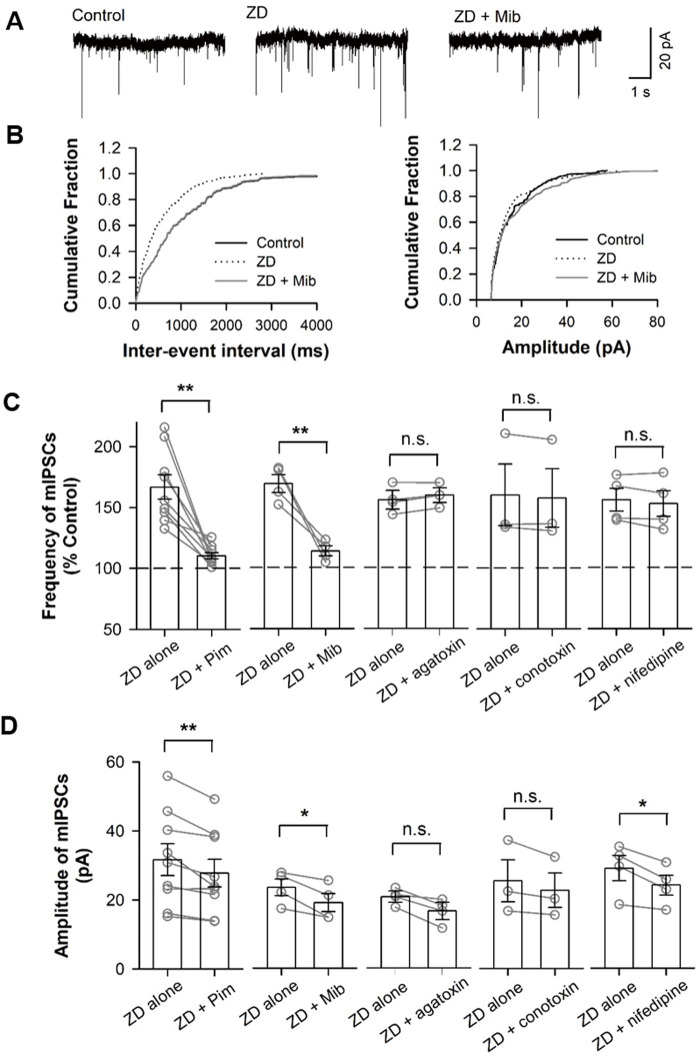
**HCN-blockade enhancement of mIPSC frequency requires Ca^2+^ influx.** (A) ZD7288 has no effect on mIPSC frequency under the condition of omitting extracellular Ca^2+^. An example trace of mIPSCs recorded in pyramidal cell under Ca^2+^-free perfusion solution (A1). The number distribution of mIPSCs (bin=60 s; A2, left), and the cumulative fraction distribution of inter-event intervals of mIPSCs (A2, right) recorded from cell in A1. The individual and grouped data showing the effect of ZD7288 on the frequency (A3, left) and amplitude (A3, right) under extracellular Ca^2+^-free condition. *n*=5 pyramidal cells. (B) Blocking Ca^2+^ channel abolishes the effect of ZD7288 on mIPSC frequency. An example trace of mIPSCs recorded in pyramidal cell (B1). The number distribution of mIPSCs (bin=60 s; B2, left), and the cumulative fraction distribution of inter-event intervals of mIPSCs before (Control), during application of ZD7288 alone (ZD7288), and during co-application of ZD7288 and Ca^2+^ channel blocker Cd^2+^ (200 µM, ZD7288+Cd ^2+^) (B2, right) recorded from cell in B1. The individual and grouped data showing the changes in mIPSC frequency (B3, left) and amplitude induced by ZD7288 alone, and co-application of ZD7288 and Cd ^2+^ (B3, right). **P<0.01, *n*=6 pyramidal cells. Calibrations: 5 s, 20 pA in A1 and B1.

It has been proved that Ca^2+^ influx is critical for the vesicle releasing at the presynaptic membrane (Kavalali, 2015). To examine whether Ca^2+^ influx is involved in ZD7288-induced facilitation of mIPSC frequency, we first examined the effect of ZD7288 under Ca^2+^-free conditions. As shown in Fig. 5A, ZD7288 failed to increase mIPSC frequency in the absence of extracellular Ca^2+^. The mIPSC frequency 12–15 min after ZD7288 application was 106.4±5.27% of control and mIPSC amplitude kept intact as well (Fig. 5A3; *P*>0.05, paired *t*-test, *n*=5 pyramidal cells from four animals). We next investigated the effect of ZD7288 in the presence of Cd^2+^ (200 µM), a Ca^2+^ channel blocker. ZD7288 significantly increased mIPSC frequency (Fig. 5B; 163.48±10.05% of control, *P*<0.01 for ZD7298 versus control, paired *t*-test). Such facilitation of mIPSC frequency was completely blocked when Cd^2+^ was added into the perfusion solution (108.0±5.82% of control; *P*<0.01 for ZD+Cd^2+^ versus ZD alone, paired *t*-test, *n*=6 pyramidal cells from three animals). Thus, these data indicate that Ca^2+^ influx is required for ZD7288-induced facilitation of mIPSC frequency.

### Voltage-gated Ca^2+^ channels are involved in facilitation of GABA release by HCN blocking

**Fig. 6.**
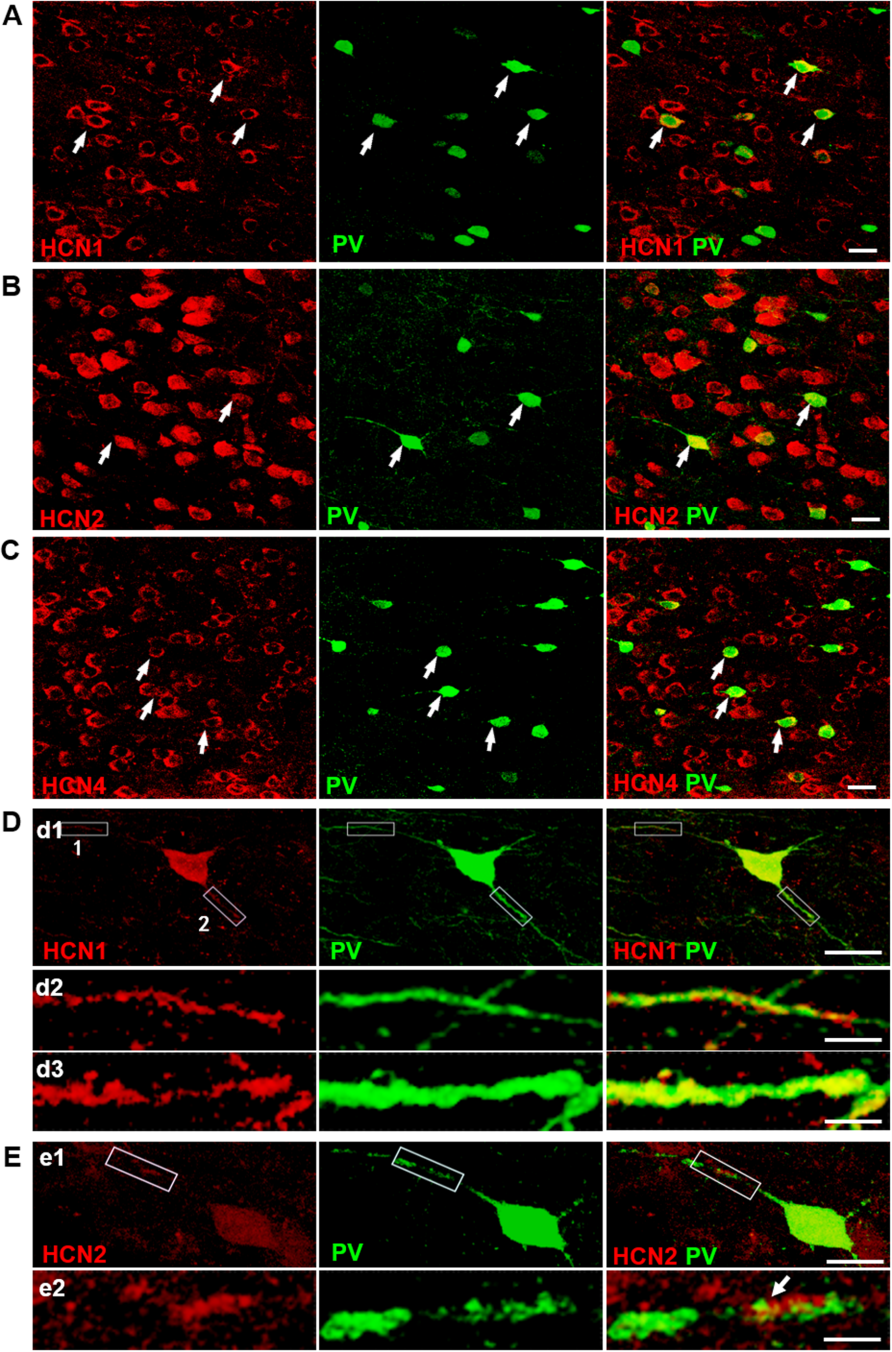
**T-type Ca2+ channel blockers occlude the increment in mIPSC frequency induced by blocking HCN channels.** (A) Representative traces of mIPSCs recorded in pyramidal cell. Holding potential: −70 mV. (B) The cumulative fraction distribution of inter-event intervals (left) and amplitude (right) of mIPSCs before (Control), during (ZD7288, 30 µM), and after co-application of ZD7288 with T-type Ca^2+^ channel selective blocker mibefradil (Mib; 10 µM) (*ZD+Mib*). (C,D) Bar graph demonstrating the effects of co-application of ZD7288 and Ca^2+^ channel blockers for T-type (pimozide, 1 µM; mibefradil, 10 µM), P/Q-type (ω-agatoxin IVA, 500 nM), N-type (ω-Conotoxin GVIA, 500 nM), and L-type (nifedipine, 2 mM) Ca^2+^ channels on the frequency (C) and amplitude (D) of mIPSCs. Open circles for individual cells and bar for grouped data. **P<0.01, paired *t*-test.

How does loss of the function in HCN channels increase Ca^2+^ influx? HCN channels open at membrane potentials more negative to −50 mV. HCN channels are also permeable to Na^+^ and K^+^ and form an inward current at rest, thereby depolarizing resting membrane potential. Thus, it is possible that a blockade of HCN channels changes resting membrane potential and alters the activity of low-voltage-activated T-type or high-voltage-activated P/Q-type, N-type, and L-type Ca^2+^ channels, resulting in Ca^2+^ influx into presynaptic terminals innervating pyramidal cells, thereby an increase in GABA release from the terminals. To test this possibility, we investigated whether the ZD7288-effect on mIPSC frequency could be occluded by Ca^2+^ channel blockers. As shown in Fig. 6C, ZD7288 had no effect on mIPSC frequency in the presence of T-type Ca^2+^ channel blocker pimozide (1 µM) (Tsubota et al., 2021) (Fig. 6B; *P*<0.01 for pimozide+ZD versus pimozide, unpaired *t*-test). Additionally, adding T-type Ca^2+^ channel blocker mibefradil (Mib; 10 µM) (Wan et al., 2020) into perfusion solution occluded the facilitation effect of ZD7288 on mIPSC frequency (*P*>0.05), while perfusing Mib (10 µM) alone had no impact on the frequency of mIPSC (*P*>0.05). But facilitation of ZD7288 on mIPSC frequency still exists in the presence of the P/Q-type Ca^2+^ channel blocker ω-agatoxin IVA (500 nM), the N-type Ca^2+^ channel blocker ω-conotoxin GVIA (500 nM), and the L-type Ca^2+^ channel blocker nifedipine (Fig. 6C; *P*>0.05, one-way ANOVA). Together, these results indicate that the blockade of HCN channels enhanced GABA release onto pyramidal cells by increasing Ca^2+^ influx through T-type Ca^2+^ channels.

### HCN channels express in parvalbumin-expressing basket cells

To examine the expression of HCN channels in GABAergic interneurons in layers 5–6 of the mPFC, we conducted double-labeling immunofluorescence staining. Four HCN channel subunits that contribute to brain HCN channels (Clapham, 1998), HCN1, HCN2, HCN3, and HCN4, have been cloned, and all four of the HCN subunits are expressed in the mammalian brain, of which HCN3 exhibits the weakest expression (Robinson and Siegelbaum, 2003). As the results presented in Figs 1 and 2 show, blockade of HCN channels induced a dramatic increase in GABA release onto pyramidal cells – most likely generated from the soma of pyramidal neurons (Soltesz et al., 1995). Interneurons expressing Ca^2+^-binding protein parvalbumin (PV) mainly innervate the soma of pyramidal cells and constitute the largest population of interneurons (∼ 50%) in the prefrontal cortex (Gonchar and Burkhalter, 1997; Markram et al., 2004; Soltesz et al., 1995; Wonders and Anderson, 2006; Kawaguchi and Kubota, 1997).

**Fig. 7.**
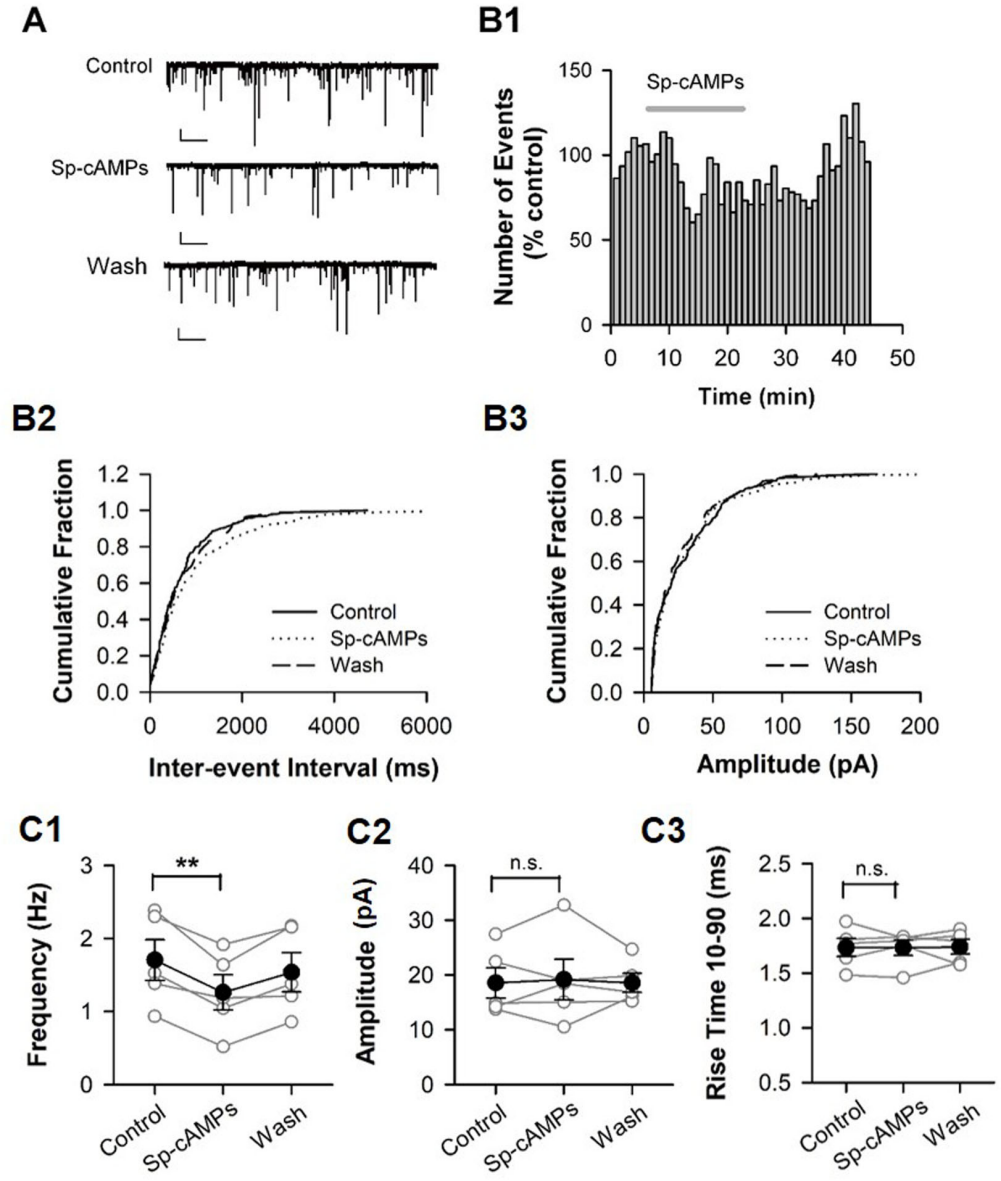
**HCN channels are present in soma and neurite of parvalbumin-expressing basket cells in layers 5–6 of mPFC.** (A–C) Microscopic confocal images showing HCN1-ir (A), HCN2-ir (B), and HCN4-ir (C) locate in PV-ir interneuron in layers 5–6 of mPFC. Double stained with HCN channels (red) and PV (green). Arrowheads indicate double-labeled cells. Scale bars: 20 µm. (D) High-magnification confocal microscopy images showing that HCN1-ir localize in the soma (d1) and along neurite (d2-d3) of PV-ir interneuron. Silhouette frame 1 and 2 in neurite (d1) is digitally magnified for a better view of neurite in (d2) and (d3), respectively. Scale bars: 20 µm in (d1) and 1 µm in (d2) and (d3). (E) High-magnification confocal microscopy images showing that HCN2-ir localize in the soma and along neurite of PV-ir interneuron. Silhouette frame in neurite (e1) is digitally magnified for a better view of neurite in (e2). Scale bars: 20 µm in (e1) and 1 µm in (e2).

We double labeled HCN1, HCN2, and HCN4 subunit with PV. Confocal microscopy images demonstrated that HCN1-ir, HCN2-ir and HCN4-ir (green) abundantly co-localized with PV-ir (red) (Fig. 7A–C). The high-magnification images depicted that both HCN1-ir and HCN2-ir was observed in neurites of PV-ir cells (Fig. 7D,E), whereas no HCN4-ir was observed (data not shown). Thus, HCN1, HCN2 and HCN4 subunit are all present in the cell bodies of PV-expressing interneurons, while only HCN1 and HCN2 express in the processing of PV-expressing interneurons in layers 5–6 of the mPFC.

## DISCUSSION

In this study, we demonstrated that HCN channels are richly present in cells expressing parvalbumin, and the pharmacological blocking of HCN channels enhances GABA release onto pyramidal neurons in layers 5–6 of mPFC through increasing Ca^2+^ influx via T-type Ca^2+^ channels.

The T-type Ca^2+^ channels are expressed in parvalbumin-expressing basket cells in the 5th and 6th cortical layers (Liu et al., 2011), suggesting that HCN channels may provide a regulatory mechanism for controlling GABA releases from GABAergic terminals. It is reported that HCN channels regulate T-type Ca^2+^ channel activity in neuronal dendrites in the hippocampus (Tsay et al., 2007) and in layer 3 glutamate terminals in the entorhinal cortex (Huang et al., 2011). Consistently, the present study demonstrated that HCN channels are present in the inhibitory presynaptic terminals of layer 5–6 basket cells in the mPFC, and constrains GABA release by restricting Ca^2+^ entry via pre-synaptic T-type Ca^2+^ channels.

The universality and nonselective cation permeability make HCN channels indispensable for cell excitability. Not only in pyramidal neurons, interneurons excitability homeostasis disorder also can trigger psychosis (Choi et al., 2020; Lin et al., 2020). Simultaneously, HCN channels engage on intracellular coupling reaction, such as alpha-2 (α_2_) adrenergic receptor, and the location of HCN channels in post-synapse membrane also facilitates their involvement in synaptic signaling (Wang et al., 2007). Along this principle, the specific expression of HCN channels in parvalbumin-expressing basket cells suggests it may be involved in GABA release (Roth and Hu, 2020). Our study is consistent with this point and proves this pathway regulates the inhibitory input of layer 5–6 pyramidal neuron in mPFC. Beside alpha-2 (α_2_) adrenergic receptor, HCN channels can also be regulated by other neurotransmitter receptors, such as dopamine receptors, through the cAMP pathway. Combining these together, some neurotransmitters would regulate GABAergic transmission by means of affecting HCN channels to control rhythmic oscillatory activity of the cortex, which is essential for brain function.

Regulation of GABA release by HCN channels is diverse, dependent on their locations and proximity to other ion channels. It is reported that, in the entorhinal cortex and globus pallidus, blockade or loss of HCN channels induces an increase in the frequency of sIPSCs and mIPSCs, with no effect on the amplitude of mIPSCs (Boyes et al., 2007; Huang et al., 2009), similar with what we found in the present study. However, some other studies showed that pharmacological blockade of HCN channels with ZD7288 results in a reduction, instead of an increase, in the frequency of sIPSCs or mIPSCs in the CA1 and DG regions of the hippocampus and in the cerebellum as well (Aponte et al., 2006; Southan et al., 2000; Lupica et al., 2001). Such discrepancy might be due to the presynaptic HCN channels exerting depolarizing influence on GABAergic terminals, and that the blockade of HCN channels inhibits neurotransmitter release via hyperpolarization. Indeed, an increase in the external K^+^ concentration from 2.5 to 5.0 mM reverses the inhibitory effect of HCN-channel blockade on mIPSC frequency (Aponte et al., 2006).

Although pyramidal cells in the mPFC receive inhibitory input from several types of interneurons (Freund and Buzsaki, 1996), the results presented here suggest that the increase in sIPSC/mIPSC frequency induced by HCN-channel blockade mainly originated from increased release of GABA from parvalbumin-expressing basket cells. First, as the source of the dominant inhibitory system with the largest population of interneurons in layers 5–6 of the prefrontal cortex (Gonchar and Burkhalter, 1997), parvalbumin-expressing basket cells should inevitably produce the largest somatically-recorded IPSCs (Markram et al., 2004; Kawaguchi and Kubota, 1997). Thus, the frequency of sIPSCs with larger amplitude should increase dramatically upon GABA release from parvalbumin-expressing basket cells in the presence of ZD7288. Indeed, our data showed that the ZD7288-induced increase in the frequency of large sIPSCs (with amplitude larger than 20 pA) was up to 183.06±21.07% of control, whereas that of all detected sIPSCs was only 135.34±10.22%. Second, ZD7288 augmented the frequency but not the amplitude or kinetics of mIPSCs (see Fig. 2E), suggesting that GABA was released tonically from the GABAergic terminals close to the soma of the pyramidal cells (Soltesz et al., 1995). Third, it has been shown that interneurons that do not express parvalbumin make synapses near the soma of pyramidal cells and utilize N-type Ca^2+^ channels in terminals for GABA release, while parvalbumin-expressing interneurons utilize P/Q-type Ca^2+^ channels in terminals for GABA release (Wilson et al., 2001; Hefft and Jonas, 2005). Our data showed that ZD7288 still increased the frequency of mIPSCs in the presence of the N-type Ca^2+^ channel blocker (see Fig. 6C). Taken together, we argue that parvalbumin-expressing basket cells produced the major contribution to the ZD7288-induced increase in spontaneous/miniature IPSCs in layer 5–6 pyramidal cells.

Parvalbumin-expressing basket cells in the cortex play an important role in adjusting the gain of synaptic input onto controlling synchronization and excitatory output of pyramidal cells, and through this mechanism, they control both the number of pyramidal cells activated and the firing frequency of the pyramidal cells (Scanziani, 2004). Pyramidal cells in layers 5–6 of the mPFC have been suggested to primarily project to subcortical regions to regulate complex motor functions, behavioral arousal and attention (Gabbott et al., 2005). The dynamic modulation of GABAergic inputs to the pyramidal cells by HCN channels in parvalbumin-expressing basket cells may contribute to the regulation of these physiological states. In addition, oscillations occurring in PFC pyramidal cells are essential for behavioral and cognitive functions (Jutras and Buffalo 2010). Coherent network oscillations, which are facilitated by GABA released onto pyramidal cells, are required for execution of cognitive functions. Abnormal γ-frequency oscillations observed in schizophrenia have been suggested as being due to a reduction in peri-somatic inhibition in PFC pyramidal cells (Lewis et al. 2011). Thus, HCN channels in parvalbumin-expressing basket cells might be a potential target for drug development for schizophrenia.

## MATERIALS AND METHODS

### Electrophysiology

Male Sprague-Dawley rats (4–5 weeks old, 80-130 g) were purchased from SLACCAS (Shanghai, China) and were kept in a 12 h light/dark cycles, and food and water were available *ad libitum*. All experiments were performed in compliance with the Guide for the Care and Use of Laboratory Animals issued by the National Institutes of Health, USA, and were approved and monitored by the Ethical Committee of Animal Experiments at the Fudan University Institute of Neurobiology (Shanghai, China). All efforts were made to minimize the number of animals used and their suffering.

Rats were anesthetized with pentobarbital sodium (40 mg/kg, i.p.) and rapidly decapitated. Brains were rapidly removed and immersed in the 0°C artificial cerebrospinal fluid (ACSF) solution containing (in mM): 119 NaCl, 2.5 KCl, 2.5 CaCl2, 1.3 MgCl2, 26.2 NaHCO3, 1.25 NaH2PO4 and 11 D-glucose. Coronal brain slices (300 µm) containing the prelimbic cortex (Paxinos and Watson, 5th edition) were cut on vibratome (Ted Pella Inc., USA) and transferred to the incubation chamber, where they were incubated in ACSF solution for at least 1 h at room temperature before recording. The ACSF solution was constantly bubbled with 95% O2–5% CO2 to maintain pH 7.4.

Whole-cell recordings were performed using standard procedures at room temperature. Brain slices were transferred to a submersion-type chamber and perfused constantly (∼2 ml/min) with ACSF. Layer 5–6 pyramidal cells were viewed using an Olympus BX51 microscope equipped with IR-DIC optics and an infrared video camera (Qimaging, Canada). Current-clamp recordings were obtained using Axon 200B amplifier, Digidata 1322 A/D converter and pClamp software (Molecular Devices, USA). Voltage- and current-clamp recordings were not corrected for the liquid junction potential. Data was discarded if Ra was altered by ∼20%. For IPSC recordings, external perfusion solution contained AMPA receptor antagonist CNQX or DNQX (20 µM) and NMDA receptor antagonist APV (50 µM). The pipette solution contained (in mM) 70 K-gluconate, 70 KCl, 20 HEPES, 0.5 CaCl_2_, 5.0 EGTA, 5 Mg-ATP, and its pH was adjusted to 7.2 with KOH. The pipette resistance, as measured in the bath, was typically 2.0–3.0 MΩ. Ion channel blockers used in this study were applied by bath perfusion for at least 10 min unless otherwise noted. To assess the role of HCN channels in GABAergic transmission, the HCN channel blocker ZD7288 was applied after 5–10 min of baseline recordings. ZD7288-induced changes in GABAergic transmission were measured in the last 3 min of the 15-min perfusion of ZD7288 unless otherwise mentioned.

### Chemicals

All reagents were purchased from the Sigma Chemical Company (St. Louis, MO, USA) with the exceptions of ZD7288 from the Tocris company (UK), ω-Agatoxin IVA, ω-Conotoxin GVIA and pimozide from Alomone Labs (Isreal). All channel blockers were prepared as concentrated stock solutions in distilled water or DMSO and either added immediately to ACSF at working concentrations or stored at −20°C for subsequent utilization.

### Immunostaining

Age-matched male Sprague-Dawley rats were anesthetized with pentobarbital sodium (40 mg/kg, i.p.), and transcardial perfusion was performed with 34°C saline (200 ml) followed by 4% ice-cold paraformaldehyde (PFA) in phosphate-buffered saline (PBS, pH 7.4). Brains were removed and were fixed for 24 h in PFA at 4°C. After that, the brains were put in 30% (w/v) sucrose solution. Coronal sections (35 µm) were cut using a cryostat (Leica CM900, Germany). Brain sections were rinsed with 0.01 M PBS and incubated in a solution of 0.5% Triton-X in PBS for 15 min, followed by normal blocking solution (goat serum, Invitrogen) for 2 h at room temperature. Sequential primary immunolabeling for HCN1, HCN2 or HCN4 was performed using anti-HCN1, HCN2 or HCN4 rabbit antibodies (1:40; Alomone Laboratories, Israel, Product# APC-056, APC-030, APC-057) (Morris et al., 2004). GABAergic neurons were labeled using anti-parvalbumin mouse antibody (PARV-19, 1:1000; Sigma-Aldrich, St Louis, MO, USA, Product# P3088). All primary antibodies were diluted in goat serum (Invitrogen) and incubated for 48 h at 4°C. After 48-h incubation, the brain sections were rinsed with PBS, and an appropriate secondary antibody was applied. Fluorescent secondary antibodies (whole IgG affinity-purified antibodies: Goat anti-rabbit FITC, Goat anti-mouse Texas Red and Goat anti-mouse FITC; all from Jackson ImmunoResearch Laboratories, West Grove, PA, USA) were applied at a 1:100 dilution in normal blocking serum for 2 h at 4°C.

### Confocal microscopy

Immunolabeled sections were examined using a confocal laser-scanning microscope system (Leica SP2, Mannheim, Germany). FITC and Texas Red fluorochromes were excited at 488 nm and 543 nm wavelengths, respectively, and the fluorescent emission was collected through BP 505–530 and BP 560–615 filters, respectively. Twelve-bit images were captured at a resolution of 1024×1024 pixels using a 20× objective and at 1024×1024 pixels with a Plan-Apochromat 63/1.4 oil-immersion. Immunoreactivity (IR) was examined under optimal resolution (small pinhole, thin optical slice, and high numerical aperture oil-immersion objective). The pinhole diameter was set to 1.5 airy unit to reduce the influence of cytoplasmic fluorescence as much as possible. Z-sectioning was performed at 0.5-µm intervals, and stacks of optical sections at the *z* axis were acquired. For comparison of the distribution of HCN1, HCN2 and HCN4 channels, each micrograph was captured using the same settings for laser power, pinhole, and photo-multiplier gain. Confocal photomicrographs were processed using Adobe Photoshop (San Jose, CA, USA). No immunolabeling was observed in control slices in which the primary antibody was omitted. The multi-tracking configuration was employed to rule out crosstalk between the fluorescent detection channels.

### Data analysis and statistics

Data are expressed as mean±s.e.m. in all cases. The frequency and amplitude of sIPSCs/mIPSCs were analyzed using the Mini Analysis software package (v8.0, Synaptosoft, Leonia, NJ, USA, http://www.synaptosoft.com). Events above fivefold baseline noise level in amplitude were detected and were used for analysis. Drug-induced changes in cumulative fractions of sIPSC/mIPSC amplitude and inter-event interval were analyzed for statistical significance using the Kolmogorov–Smirnov (K-S) test (Mini Analysis v8.0) and a conservative critical probability level of *P*<0.01. Grouped data were analyzed using paired or unpaired *t*-test for two-group comparison, one-way ANOVA for multi-group comparison, and a critical probability of *P*<0.05 (STATISTICA 6.0, USA).
